# Bitter taste receptors along the gastrointestinal tract: comparison between humans and rodents

**DOI:** 10.3389/fnut.2023.1215889

**Published:** 2023-08-30

**Authors:** Maria Descamps-Solà, Adrià Vilalta, Florijan Jalsevac, M. Teresa Blay, Esther Rodríguez-Gallego, Montserrat Pinent, Raúl Beltrán-Debón, Ximena Terra, Anna Ardévol

**Affiliations:** Departament de Bioquímica i Biotecnologia, Universitat Rovira i Virgili, MoBioFood Research Group, Tarragona, Spain

**Keywords:** extraoral TAS2R, taste sensing, orthology, nutrient sensors, gut

## Abstract

For decades bitter taste receptors (TAS2R) were thought to be located only in the mouth and to serve as sensors for nutrients and harmful substances. However, in recent years Tas2r have also been reported in extraoral tissues such as the skin, the lungs, and the intestine, where their function is still uncertain. To better understand the physiological role of these receptors, in this paper we focused on the intestine, an organ in which their activation may be similar to the receptors found in the mouth. We compare the relative presence of these receptors along the gastrointestinal tract in three main species of biomedical research (mice, rats and humans) using sequence homology. Current data from studies of rodents are scarce and while more data are available in humans, they are still deficient. Our results indicate, unexpectedly, that the reported expression profiles do not always coincide between species even if the receptors are orthologs. This may be due not only to evolutionary divergence of the species but also to their adaptation to different dietary patterns. Further studies are needed in order to develop an integrated vision of these receptors and their physiological functionality along the gastrointestinal tract.

## 1. Introduction

Animals constantly need to consume energy-providing foods and micronutrients in order to maintain optimal functionality. When absorbing nutrients, their senses promote satiety and maintenance of good homeostatic control. Specifically, taste sensing promotes adequate digestive and hormonal responses (1). This capability also facilitates recognition of beneficial or potentially harmful food components (2, 3). The first step in taste sensing takes place within the oral cavity via the recognition of nutrients by specific receptors (2, 4). However, some of these receptors have also been described in extraoral tissues such as the gastrointestinal tract, the respiratory system, and the reproductive organs (1, 2, 5).

Taste receptors (TASRs) share their role in detecting food components. However, they are structurally and functionally different. Salt and sour taste are detected by specialized ion channels (6) while sweet, umami and bitter tastes are detected by specific G-protein-coupled receptors. The recognition of taste in the oral cavity is mediated through taste organs named taste buds (7). Moreover, each of the five basic tastes (salty, sour, sweet, umami, and bitter) (8, 9) are detected by taste cells, which are divided into three types: types I, II, and III (8, 9). Type II cells are further divided into sweet, umami, and bitter cells (9, 10). Here, the corresponding receptors interact with carbohydrates, proteins, lipids, and other tasting substances to promote taste perception (6, 11).

TASRs can be divided into two gene families: the taste-1 receptor family (TAS1R) and the taste-2 receptor family (TAS2R). TAS1R comprises three members: TAS1R1, TAS1R2, and TAS1R3 (12). TAS1Rs form oligomeric complexes among the various subunits that enable them to detect sweet- and umami-tasting L-amino acids. The umami receptor complex is made up of TAS1R1 and TAS1R3 subunits, while the sweet receptor complex is made up of TAS1R2 and TAS1R3 subunits (12). TAS2R comprises the receptors in charge of bitter taste sensing. Unlike TAS1R, this family comprises multiple different receptors whose number varies from species to species (3, 12). This wide diversity in TAS2R, their variability among species, and a lack of information on their role beyond oral taste perception highlight the need to analyse them to learn about their functions and regulation. In this paper we address this issue by comparing their relative presence along the gastrointestinal tract in three widely used species at the preclinical and clinical levels (mice, rats and humans). In this context, we are not addressing the sensitivity of each type of bitter taste receptor to ligand stimulation, as this constitutes a separate extensive topic. It is noteworthy that certain TAS2R receptors display high promiscuity, whereas others exhibit greater selectivity, further demonstrating variations in specificities across different species (13, 14). Although only partial information is available on this aspect, some intriguing reviews have been published, offering insights into the existing data (15, 16).

## 2. Bitter taste receptors (tas2r)

Receptors in the TAS2R family, i.e., bitter taste receptors, belong to a diverse group of GPCRs expressed in the tongue and, specifically, in *vallate papilla* cells (VP) (9). As their name suggests, their main role is to sense bitter components in food. This is a defence mechanism since, by sensing bitter, a food’s potentially toxic components can be detected (4) and their ingestion prevented, thus avoiding potential harmful effects. Not all bitter compounds are toxic, however, as they can also be found in high concentrations and with health benefits in foods of vegetal origin (3, 4, 12).

### 2.1. TAS2R action mechanism on the tongue

**Figure 1:**
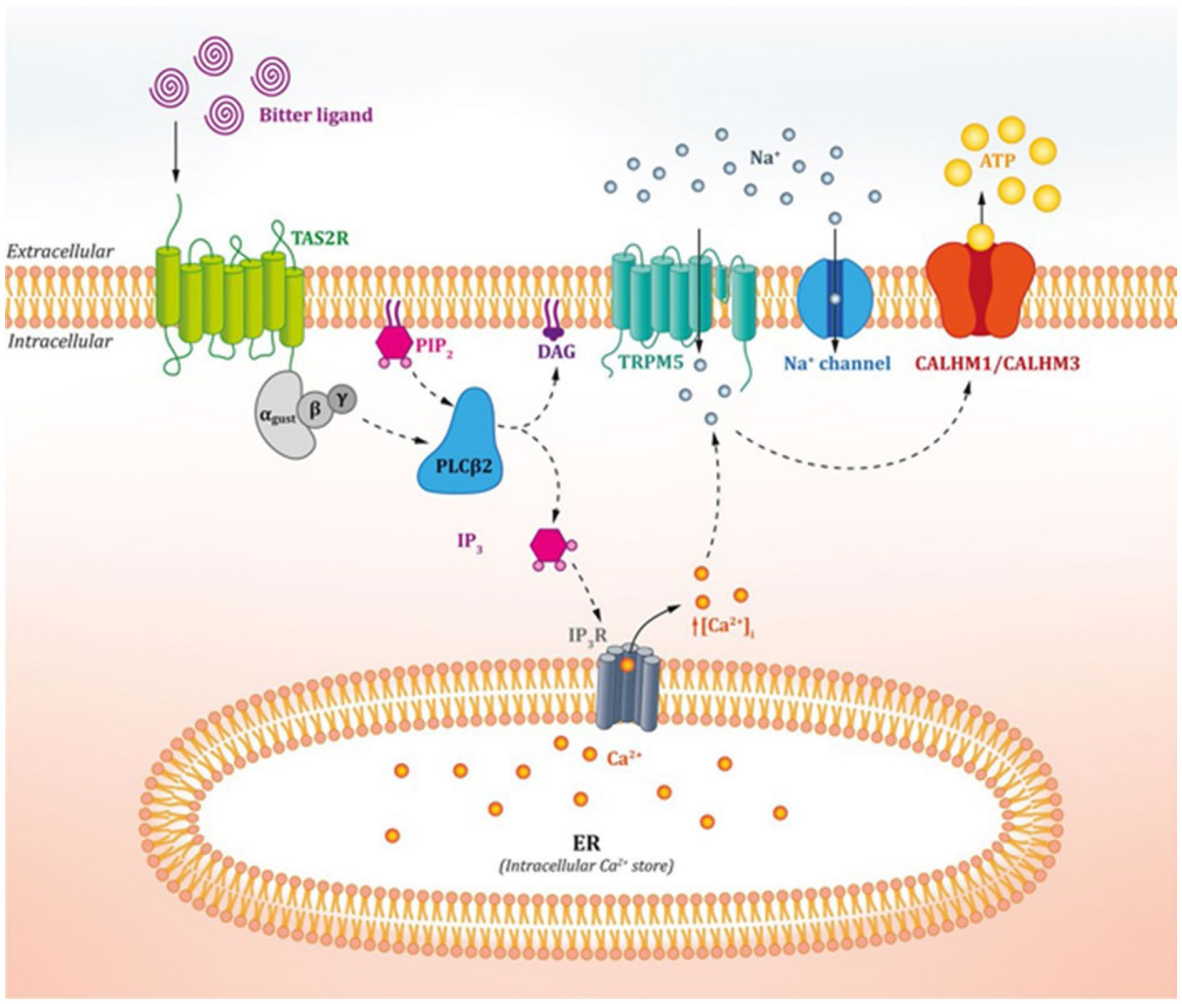
Action mechanism of oral bitter taste receptors. Figure extracted from Tuzim and Korolczuk (13).

Although different taste receptors detect nutrients and non-nutrients on the tongue, bitter, sweet, and umami receptors work in a similar way. The taste pathway (Figure 1) begins with the binding of the taste ligand to the specific receptor complex. This activates the G-protein-coupled receptor (9) and leads to dissociation of the GTP-bound heterotrimeric G proteins. These proteins work as a molecular switch inside cells and are made up of three G-subunits, i.e., the α-, β-, and γ-subunits (14). In the case of bitter taste receptors, α-gustducin was the first and is currently the best-characterized G protein in taste cells (1, 15). These proteins activate the membrane-bound phospholipase C2 (PLCβ2), thus inducing the production of inositol-1,4,5-thriphosphate (IP3) and diacylglyceride (DAG) (1, 3). IP3 binds to its receptor located in the membrane of the endoplasmic reticulum, thus activating calcium channels, and leading to an increase in intracellular calcium ions (3, 15). The calcium ions and DAG activate the transient receptor potential melastatin channel subtype 5 (TRPM5), causing depolarization and therefore the activation of voltage-gated Na + channels (VGNC). This activates CALHM1/CALHM3 channels, which induces the release of ATP as a neurotransmitter, thus activating purinergic receptors on afferent nerve fibres and leading to taste perception (1, 3, 9, 16).

### 2.2. Extraoral bitter taste receptors

In the last decade, several studies have reported that, as well as orally, bitter taste receptors are expressed in tissues such as the gastrointestinal tract, the lungs, the skin and the reproductive system (2, 9, 13, 17–19). Since in these extraoral locations sensing taste is not required, the locations must be involved in other physiological events (20, 21).

The morphology of extraoral bitter receptors resembles that of oral receptors, which enables them to detect the same ligands despite their different physiological role (9). Recent studies suggest that bitter taste receptors play a role in, for example, inflammation, bronchial relaxation in the respiratory system, and control of enteroendocrine systems (3, 12, 22). Despite recent research, these extraoral receptors are still a novel topic and much is still unknown, though their action mechanism is believed to have similarities with that of oral receptors. An increase in calcium levels in the enteroendocrine cells has been shown to lead to hormone secretion, which modulates physiological functions in the gastrointestinal tract such as hunger regulation and metabolic homeostasis (15, 19).

### 2.3. TAS2R diversity among species

Since bitter taste receptors play such an important role in an organism’s protective and homeostasis system, unsurprisingly they can be found in all animal species from insects to primates (23). Though they are found throughout the animal kingdom, the number of different bitter taste receptors varies between species. Though evolutionary separate and specialized to the requirements of the species, the taste-sensing pathways of insects and mammals are remarkably similar. *Drosophila* (commonly known as fruit flies) have specialized taste sensilla on their legs (24). Since fruit flies typically walk on the fruits they feed on, it is advantageous for them to sense their nutritional components before ingesting them. The typical taste receptors discussed earlier are not present in these animals, which sense most bitter compounds via specialized L2 sensilla that are also stimulated by salts (24).

The taste receptors of vertebrates have developed relatively recently. Their absence in cartilaginous fish suggests that they developed after the evolutionary split from teleostean fish, which do have bitter taste receptors (25). However, this is a theoretical explanation and other scenarios have been proposed. Despite their uncertain origin, the diversity of these receptors between species is now clear. In fact, their numbers vary dramatically from one species to another – from lost taste-sensing receptors in penguins (26) to 80 genes reported in coelacanths (27). Explaining such a large difference between species has been the focus of several recent studies in which a clear connection has been made between a species’ specific diet and its number of bitter taste receptors (28). Research has shown that carnivorous animals do not need such a wide range of taste receptors and so have fewer genes for this type of receptor (29, 30). On the other hand, since plants carry more toxic compounds than meat, it is logical that herbivores possess more sensitive systems in order to detect possible toxins (31).

Most of our knowledge of bitter taste receptors comes from human and animal studies (primarily on mice and rats). In humans, 25 functional genes and 11 pseudogenes encoded in chromosomes 5, 7 and 12 have been described (32–34). Mice have 36 functional receptors (4), which are encoded in chromosomes 2, 6, and 15 (33), and 6 pseudogenes (30). Rats possess 37 subtypes of bitter taste receptors (4), which are encoded in chromosomes 2, 3, and 4 (33) and possess 5 pseudogenes (30).

Here a question arises, however: if the fundamental role of bitter taste receptors is to detect bitter compounds, why are there so many different receptors? The answer could lie in their specific nature. Within this broad range of receptors, some are more specific and are activated by just a few ligands, while others are a target for many different compounds (4, 35). At least in humans, TAS2Rs have been divided into four groups: (i) receptors that detect a broad spectrum of ligands, (ii) more selective receptors that detect just a few ligands, (iii) receptors that lie somewhere between these first two groups, and (iv) receptors that recognize specific chemical motifs (35). Moreover, since rodents have significantly more functional receptors than humans, they may have a wider bitter-sensing spectrum than humans (4, 12).

### 2.4. Comparison of TAS2R orthology between species

Several studies have evaluated the evolutionary pathway followed by TAS2Rs in humans, rats, mice, and other species (4, 36, 37). To establish the various orthologs among different species, we combined data from the phylogenetic trees based on similarity in the alignment of amino acid sequences (the Neighbour-Joining Method) (Table 1). In this review only the receptors from humans, rat and mice were considered.

**Table 1:** Comparison between the bitter taste receptors of human, rat (human and mouse nomenclatures), and mouse structural orthologs.

Human	Rat		Mouse
	Human nomenclature	Mouse nomentature	
TAS2R1	rTas2r1	rTas2r119	mTas2r119
TAS2R3	rTas2r37	rTas2r137	mTas2r137
TAS2R4	rTas2r16	rTas2r108	mTas2r108
TAS2R5	X	X	X
TAS2R7	rTas2r6	rTas2r130	mTas2r130
TAS2R8	X	X	X
TAS2R9	X	X	X
TAS2R10	rTas2r18	rTas2r104	mTas2r104
	rTas2r9	rTas2r105	mTas2r105
	rTas2r30	rTas2r106	mTas2r106
	rTas2r4	rTas2r107	mTas2r107
	rTas2r5	rTas2r114	mTas2r114
TAS2R13	–	rTas2r13	X
	rTas2r8/rTas2r21	rTas2r102	mTas2r102
	rTas2r7	rTas2r121	mTas2r121
	rTas2r24	rTas2r124	mTas2r124
TAS2R14	rTas2r23	rTas2r103	mTas2r103
	rTas2r26	rTas2r109	mTas2r109
	rTas2r10	rTas2r110	mTas2r110
	rTas2r31	rTas2r113	mTas2r113
	X	X	mTas2r115
	rTas2r32	rTas2r116	mTas2r116
	rTas2rr17	rTas2r117	mTas2r117
	rTas2r2	rTas2r123	mTas2r123
	rTas2r25	rTas2r125	mTas2r125
	rTas2r29	rTas2r129	mTas2r129
	rTas2r40	rTas2r140	mTas2r140
TAS2R16	rTAS2R3	rTas2r118	mTas2r118
	rTas2r34	rTas2r134	mTas2r134
	rTas2r43	rTas2r143	mTas2r143
TAS2R19			
TAS2R20/TAS2R49	rTas2r36	rTas2r136	mTas2r136
TAS2R30/TAS2R47	rTas2r20	rTas2r120	mTas2r120
TAS2R31/TAS2R44			
TAS2R38	rTas2r38	rTas2r138	mTas2r138
TAS2R39	rTas2r39	rTas2r139	mTas2r139
TAS2R40	rTas2r44	rTas2r144	mTas2r144
TAS2R41	rTas2r12	rTas2r126	mTas2r126
TAS2R42	rTas2r13	rTas2r145	mTas2r131
TAS2R43	–	rTas2r136 and rTas2r120	mTas2r136 and mTas2r120
TAS2R45			
TAS2R46			
TAS2R50			
TAS2R60	rTas2r35	rTas2r135	mTas2r135
X	X		mTas2r122
?	rTas2r33*	–	mTas2r133*
X	–	mTas2r7	X

X means that there are no orthologs in the species; – means that the nomenclature is nonexistent; and ? means that no information exists on the presence of the ortholog in that species. Based on (4, 36, 37).

As well as the scarcity of reports addressing TAS2R function and localization, a further challenge when comparing TAS2Rs among different species is that two nomenclatures exist for rats. One of these is similar to that used for human bitter receptors, while the other is similar to that used for mouse receptors. However, the one most widely used today is mouse nomenclature thus, the human nomenclature will not be utilized in the reminder of the review. Table 1 shows the nomenclatures used and compares the structural orthologs of these three species.

Table 1 shows that some receptors possess only one ortholog in each species, thus indicating a similar evolutionary pathway. Due to the difference in the number of receptors found in different species, some receptors present one ortholog in humans while in rodents the receptor diverges into several subtypes. This phenomenon can also be found in the opposite direction, i.e., one ortholog in rodents and several subtypes in humans, though human orthologs are fewer and less frequent. The remaining receptors diverged in the early stages of evolution and can be found in only one species with no similar orthologs in the other species analysed.

For comparison between phylogenetic studies, the data presented were robust in most cases. However, some receptors, i.e., TAS2r7l (4), TAS2R13 (4), and rT2R33/mTas2r133 (36) are described only in one study showing some discrepancies.

## 3. The role of bitter taste receptors in the gastrointestinal tract

Since the gastrointestinal tract (GIT) is the primary point of interaction for ingested nutrients, it is an important organ for metabolic regulation and whole-body function (38). Luminal contents can transit over the GIT via peristaltic motions, whereas smooth muscle segmentation allows for appropriate contact time with the epithelial mucosal area, thus enabling proper absorption. In the GIT, nutrients are digested by digestive enzymes and absorbed (39). Nutrient interactions with the small and large intestine produce feedback responses that, for example, slow stomach emptying, cause satiation, or lower postprandial hyperglycaemia excursions.

As mentioned earlier, TAS2R receptors are expressed in some cells of the GIT. Bitter components of ingested food bind to these receptors and begin a signalling pathway that is believed to be shared at least partially with the lingual receptors. However, in the GIT, the physiological response is completely different from the oral response, where the signal is sent to the brain and interpreted as bitter taste.

### 3.1. TAS2R regulate the GIT barrier and communication with the body

One group of intestinal cell types that express TAS2Rs are the enteroendocrine cells, which act as nutrient sensors and control the release of gut peptides called enterohormones (16, 40). Activation of this pathways leads to the control of food intake and metabolic homeostasis (3, 21). Depending on the intestinal segment, bitter taste receptors can modulate the secretion of different enterohormones through α-gustducin. The main enterohormones involved are ghrelin (41, 42), glucagon-like peptide 1 (GLP-1) (43–45), peptide YY (PYY) (43), and cholecystokinin (CCK) (20, 44, 45). The main effects of some of these enterohormones are exerted in the vague nerve, with an increase in the sensation of satiation and the motility of the gastrointestinal tract (16). Enterohormones also play a crucial role in coordinating digestion, gastric emptying, absorption, food output, and overall metabolic homeostasis (46).

Tuft cells are shown to express bitter, sweet and umami receptors and act as chemosensory epithelial cells. These cells mainly detect helminths and protozoa, and their activation leads to a broad secretion of cytokines such as interleukin 25, prostaglandin E2 and D2, cysteinyl leukotriene C4, acetylcholine, thymic stromal lymphopoietin, and β-endorphins, some of which possess immunomodulatory effects. Together, these effects enhance the immune response and drive the excretion of parasites (45, 47).

Other authors have reported that Goblet and Paneth cells found in jejunal crypts also express Tas2R. The activation of these receptors by bitter molecules can modulate the mRNA expression of antimicrobial peptides such as α-defensin, β-defensin and regenerating family member 3 alpha (REG3A), mucus protein-encoding genes and mucins, and chemokines such as CCL3 and CCL4. Simultaneously, treatment with bitter substances activates the NRF2-mediated oxidative stress response, the unfolded protein response, integrin-linked kinase (ILK), AMP activated protein kinase (AMPK), p38 mitogen-activated protein kinases (p38 MAPK), and phospholipase C signalling while downregulating the integrin and Eukaryotic Initiation Factor 2 (EIF2) signalling pathways. All these effects on mucus production and innate immune response may be beneficial in the modulation of metabolic homeostasis via control of bacterial growth (22). In fact, distinct microbiota traits have been associated with genetic variants of TAS2R38 (54).

Recent research has unveiled the immunomodulatory potential of TAS2R activation in immune cells, with Tas2R agonists demonstrating anti-inflammatory effects against various inflammatory airway diseases, such as asthma (55–57), and bone inflammation in orthopaedic disorders (58). Notably, Tran et al. (59) identified the expression of T2R38 in human peripheral mononuclear blood cells (PMBCs), encompassing lymphocytes, monocytes, and granulocytes. Additionally, Grassin-Delyle et al. (60) discovered TAS2R subtypes that are likely involved in inhibiting lipopolysaccharide (LPS)-induced cytokine production in human lung macrophages, aside from their role as bronchodilators on smooth muscle cells. In human gingival fibroblasts, TAS2R16 activation was found to inhibit the production of LPS-induced proinflammatory cytokines by repressing cAMP levels. Furthermore, TAS2R16 demonstrated the ability to antagonize nuclear factor kappa-light-chain-enhancer of activated B cells (NF-KB) signaling by impeding its translocation to the nucleus (61). The diverse roles of bitter taste receptors in managing inflammation, infection, and modulating the epithelial barrier and inflammatory response are becoming evident through these findings.

Despite the significant progress in understanding the properties of extra-oral TAS2Rs, the link between TAS2R activation and the development of metabolic syndrome remains not completely understood (62, 63). Encouraging preclinical studies on animal models of obesity and inflammation have demonstrated that oral administration of ARD-101, a potential TAS2R agonist, led to reduced food intake, decreased body weight, and downregulated inflammatory cytokines through their effects at the gut level (64). Currently, the potential of ARD-101 is being evaluated in phase 2 trials involving patients with metabolic and inflammatory disorders (64). Furthermore, Kok et al. (65) highlighted that activating Tas2r108 in the gut can remodel enteroendocrine hormone release and bile acid metabolism, ameliorating multiple features of metabolic syndrome. This suggests that targeting extraoral bitter taste receptors could hold promise in managing metabolic diseases.

Overall, the emerging evidence suggests that human intestinal bitter taste receptors play crucial roles in regulating innate immune responses and metabolic regulators in the context of obesity. Further investigations in this field can open up new avenues for therapeutic interventions targeting TAS2R activation in immune and metabolic disorders.

## 4. Bitter taste receptor expression through the GIT

Although overall understanding of the TAS2R expression profile along the gastrointestinal tract is still incomplete, it is now clear that the distribution and abundance of bitter taste receptors along the GI system is complex (21). Numerous TAS2R have been observed in the intestinal tissues of humans and rodents with noticeable differences in expression profiles in different parts of the GIT (2, 36, 49).

The different number of receptors between humans and rodents suggest that the expression profile will differ between species. Moreover, the intestine is more restrictive than the oral cavity (specifically the VP on the tongue) with regard to the expression of TAS2R subtypes. All the above leads to a unique expression profile for each species and each intestinal segment, which must be compared if we are to acquire knowledge about their role in the GIT.

Since the expression of TAS2R subtypes is significantly lower in extraoral tissues than in the VP of the tongue (49), it is more difficult to study the expression profile of extraoral bitter taste receptors. TAS2Rs can be divided into three groups according to their expression profile: TAS2Rs that are expressed in all types of tissues, TAS2Rs that are expressed only in the gastrointestinal tract, and TAS2Rs that are expressed only in a specific segment of the GIT or specific organs (49).

In this paper we have evaluated several studies conducted on different intestinal segments and with different methodologies (e.g., relative expression studies, DNA amplification, and transcriptomics) to establish the presence of TAS2Rs in humans, rats and mice. In rodent studies, only reports with similar experimental conditions (quantitative PCR, DNA amplification, similar initial cDNA concentration) were used to combine multiple data. Genomic databases such as Rat Genome Database^1^ and Mouse Genome Informatics^2^ were consulted to verify the experimental data with transcriptomic experiments. For humans, quantitative PCR and DNA amplification studies were consulted along with tissue microarray data from the publicly available The Human Protein Atlas.^3^

### 4.1. TAS2R expression in the GIT of mice

Only a few authors have quantified the expression of all Tas2r subtypes genes along the entire mouse GIT (36, 49–52). Since the GIT begins at the mouth, every Tas2r is expressed in the tongue, though not all at the same level, with a 100-fold difference between the lowest and highest expression levels. Globally, the expression of these receptors in the tongue is much lower than in regular genes. No information is available about the presence of bitter receptors in the walls of the oral cavity since studies focus mainly on the expression of Tas2r in the tongue.

Table 2 summarizes the receptors whose expression has been reported and the intestinal segment in which it was detected (36, 49–52). Receptors whose expression has never been described are excluded from this table. Some of the data analysed were inconsistent between studies and in case of contradiction, detection of expression prevailed over non-detection. This criterion was established because of the particularly low expression of bitter taste receptors and because the lack of expression in certain receptors should not always be interpreted as repressed expression but as yet undetected expression.

**Table 2:** TAS2R expression profile along the GIT in mice.

GIT section	mTas2r#																
	108	109	113	115	117	118	119	125	126	129	131	134	135	137	138	140	143
Stomach	+	+	+	+					+				+	+		+	+
Duodenum	+						+		+			+	+	+	+		+
Jejunum	+						+		+				+	+	+		+
Ileum	+						+		+	+			+	+	+		+
Caecum	+								+				+	+	+		+
Colon	+		+		+	+	+	+	+		+		+	+	+	+	+

The “+” signs mean that expression has been reported while the blanks mean that no information is available. Colour intensity correlates with relative expression intensity. The data were obtained from (36, 49–52).

The bitter taste receptors expressed throughout the GIT are mTas2r108, mTas2r126, mTas2r135, mTas2r137, and mTas2r143 (49). In addition to the above receptors, in the stomach we also find mTas2r109, mTas2r113, mTas2r115, and mTas2r140 (49). The duodenum, jejunum, and ileum share a similar expression profile and additionally express mTas2r119 (49, 51, 52) and mTas2r138 (36, 50, 52). However, some differences exist between segments of the small intestine since the duodenum also expresses mTas2r134 (36) and the ileum also expresses mTas2r129 (49). Interestingly, of all segments of the intestine, the colon was where the highest number of receptors was expressed as it shared receptors such as mTas2r113, mTas2r119, mTas2r138, and mTas2r140 as well as mTas2r117, mTas2r118, mTas2r125, and mTas2r131 (these latter receptors were expressed only in this segment) (49, 52).

Data show that of the receptors expressed throughout the GIT, mTas2108, mTas2r126, mTas2r135, mTas2r137, and mTas2r143 possess the highest expression levels of all bitter receptors (49). These are followed by specific receptors such as mTas2r119 and mTas2r138 (49, 52) and finally those found only in specific parts of the intestine where expression is extremely low (49, 52).

### 4.2. TAS2R expression in the GIT of rats

Little is known about Tas2r expression in the intestine of rats since few studies have evaluated the expression of bitter taste receptors in segments of their intestine (36, 51, 53). These studies focused mainly on the stomach (antrum and fundus), duodenum and colon. Table 3 summarizes the data collected on receptors with their reported expression. Here only mouse nomenclature is considered.

**Table 3:** TAS2R expression profile along the GIT in rats.

GIT section	rTas2r#																			
	102	104	105	106	107	108	109	110	114	118	119	121	123	126	130	134	135	138	143	144
Fundus	+		+		−	+		−	+	+	+	+	+	+	+	+		+		
Antrum	+		+		−	+		+		+	+	+	+	+	+	+		+		
Duodenum	+	+	+	+	+	+		−	+	+	+	+	+	+	+	+	+	+	+	+
Colon						+	+				+									

The + signs mean that expression has been reported, the − signs mean that no expression is detected, and the blanks mean that no information is available. The data were obtained from (36, 51, 53).

The data indicate that the stomach can express some bitter taste receptors [even differentially between the fundus and the antrum (rTas2r110 and rTas2r114)] though the receptors are mainly expressed in both parts of the stomach. In the duodenum, the number of receptors expressed is higher since, in addition to those expressed in the stomach, the expression of rTas2r104, rTas2r106, rTas2r107, rTas2r135, rTas2r143, and rTas2r144 has been reported (36, 51). In the colon, the number of receptors found is very low mainly due to a lack of research conducted on this intestinal segment. Information on the jejunum or ileum is not available (53).

With regard to transcriptomic validation using RGD for bitter taste receptors, levels of expression in intestinal segments were not always available or were below the cut-off value. As we could not confirm the results obtained with transcriptomics assays, we have not included them in our report.

Taking into account the limitations associated with data availability, we found a clear difference in the expression profiles of the two rodent species. In mice, the stomach has a higher number of expressed receptors than the duodenum while in rats the reverse is true. Also, in mice the colon is the intestinal segment with the highest number of receptors whereas, at least from the existing data, in rats it is the segment with the lowest number. Surprisingly, despite their evolutionary proximity, rats express a higher number of receptors in the stomach and duodenum than mice do.

### 4.3. TAS2R expression in the GIT of humans

TAS2R expression in the human intestine is more widely reported than it is in the intestine of rodents. The data analysed were obtained from RNA-sequencing studies and PCR analysis. For most receptors the data are consistent between these methodologies. In some cases, however, discrepancies arise mainly in relation to the detection level. Transcriptomics enables the detection of multiple gene transcripts at the same time but lower expressed genes are not always reported. Highly expressed receptors are often reported in all databases consulted, thus validating the expression results. On the other hand, RT-PCR is a highly specific technique that enables detection at low concentrations. When we combined the two data sources, therefore, we found that some genes detected by RT-PCR were not detected by transcriptomic analysis.

In humans, several receptors, such as TAS2R4, TAS2R5, TAS2R14, andTAS2R20/49, are expressed through the intestine (1, 22, 53–56). Table 4 summarizes the TAS2Rs that have so far been detected in the human GIT. Tissue specificity applies to TAS2R1 (53), TAS2R42, TAS2R45, TAS2R46, TAS2R50, and TAS2R60 (56), which are expressed only in the colon, and to TAS2R7 and TAS2R8, which have been observed only in the jejunum (22). Other receptors expressed are TAS2R3, TAS2R10, TAS2R19, TAS2R31/44, TAS2R38, and TAS2R43 in the jejunum, ileum and colon (22, 54, 56). In addition, TAS2R10, TAS2R19, and TAS2R31/44 are expressed simultaneously in the oesophagus and TAS2R38 is also expressed in the duodenum (54). Several other receptors are expressed in different parts of the intestine, including receptors TAS2R13, TAS2R30, TAS2R39, and TAS2R40, which are found in the jejunum and colon (1, 22, 56).

**Table 4:** TAS2R expression profile along the GIT in humans.

GIT section	hTAS2R#																					
	1	3	4	5	7	8	10	13	14	19	20	30	31	38	39	40	42	43	45	46	50	60
Oesophagus	−	−	+	+	−	−	+	−	+	+	+	−	+	−	−	−	−	−	−	−	−	−
Stomach	−	−	+	+	−	−	+	−	+	+	+	−	+	−	−	−	−	−	−	−	−	−
Duodenum	−	−	+	+	−	−	−	−	+		+	−	−	+	−	−	−			−	−	−
Jejunum	−	+	+	+	+	+	+	+	+	+	+	+	+	+	+	+	−	+		+	−	−
Ileum	−	+	+	+	−	−	+	−	+	+	+	−	+	+	−	−	−	+	−	−	−	−
Colon	+	+	+	+	−	−	+	+	+	+	+	+	+	+	+	+	+	+	+	+	+	+

The “+” signs mean that expression has been reported, the − signs mean that no expression is detected, and the blanks mean that no information is available. The data were obtained from Human Protein Atlas (54), Genome Database (55) and Bertran et al. (1), Kaji et al. (53), Liszt et al. (22), and Rozengurt et al. (56).

As in mice, the human colon has the highest number of receptors, with almost all receptors being expressed there (Table 4). The colon expresses several receptors that are shared with other intestinal segments but also has tissue specificity as for T2R1, T2R42, T2R50, and T2R60 (56, 57). One probable explanation for this is that, in the colon, microbiota may generate bitter ligands associated with the fermentation process of molecules. Another feasible explanation is that the greater availability of human colon samples means that more data are present in the literature.

### 4.4. Comparison of human and rodent TAS2R expression

To better understand the role of these receptors in the GIT, we compared the different GIT locations of rodents and humans and paired the receptors according to the evolutionary pathway. In Table 5 we consider only those receptors whose expression is reported and included in Tables 2–4.

**Table 5:** Comparison of TAS2R expression profiles of humans and rodents.

Stomach			Duodenum			Jejunum			Ileum			Colon		
Human	Rat	Mice	Human	Rat	Mice	Human	Rat	Mice	Human	Rat	Mice	Human	Rat	Mice
T2R1	rT2r119	mTAS2r119	T2R1	rT2r119	mTAS2r119	T2R1	rT2r119	mTAS2r119	T2R1	rT2r119	mTAS2r119	T2R1	rT2r119	mTAS2r119
T2R3	?	mTAS2r147	T2R3	?	mTAS2r147	T2R3	?	mTAS2r147	T2R3	?	mTAS2r147	T2R3	?	mTAS2r147
T2R4	rTAS2r108	mTAS2r108	T2R4	rTAS2r108	mTAS2r108	T2R4	rTAS2r108	mTAS2r108	T2R4	rTAS2r108	mTAS2r108	T2R4	rTAS2r108	mTAS2r108
T2R5	?	?	T2R5	?	?	T2R5	?	?	T2R5	?	?	T2R5	?	?
T2R7	rTAS2r130	mTAS2r130	T2R7	rTAS2r130	mTAS2r130	T2R7	rTAS2r130	mTAS2r130	T2R7	rTAS2r130	mTAS2r130	T2R7	rTAS2r130	mTAS2r130
T2R8	?	?	T2R8	?	?	T2R8	?	?	T2R8	?	?	T2R8	?	?
T2R10	rTAS2r104	mTAS2r104	T2R10	rTAS2r104	mTAS2r104	T2R10	rTAS2r104	mTAS2r104	T2R10	rTAS2r104	mTAS2r104	T2R10	rTAS2r104	mTAS2r104
	rTAS2r105	mTAS2r105		rTAS2r105	mTAS2r105		rTAS2r105	mTAS2r105		rTAS2r105	mTAS2r105		rTAS2r105	mTAS2r105
	rTAS2r106	mTAS2r106		rTAS2r106	mTAS2r106		rTAS2r106	mTAS2r106		rTAS2r106	mTAS2r106		rTAS2r106	mTAS2r106
	rTAS2r107	mTAS2r107		rTAS2r107	mTAS2r107		rTAS2r107	mTAS2r107		rTAS2r107	mTAS2r107		rTAS2r107	mTAS2r107
	rTAS2r114	mTAS2r114		rTAS2r114	mTAS2r114		rTAS2r114	mTAS2r114		rTAS2r114	mTAS2r114		rTAS2r114	mTAS2r114
T2R13	rTAS2r102	mTAS2r102	T2R13	rTAS2r102	mTAS2r102	T2R13	rTAS2r102	mTAS2r102	T2R13	rTAS2r102	mTAS2r102	T2R13	rTAS2r102	mTAS2r102
	rTAS2r121	mTAS2r121		rTAS2r121	mTAS2r121		rTAS2r121	mTAS2r121		rTAS2r121	mTAS2r121		rTAS2r121	mTAS2r121
	rTAS2r124	mTAS2r124		rTAS2r124	mTAS2r124		rTAS2r124	mTAS2r124		rTAS2r124	mTAS2r124		rTAS2r124	mTAS2r124
T2R14	rTAS2r103	mTAS2r103	T2R14	rTAS2r103	mTAS2r103	T2R14	rTAS2r103	mTAS2r103	T2R14	rTAS2r103	mTAS2r103	T2R14	rTAS2r103	mTAS2r103
	rTAS2r109	mTAS2r109		rTAS2r109	mTAS2r109		rTAS2r109	mTAS2r109		rTAS2r109	mTAS2r109		rTAS2r109	mTAS2r109
	rTAS2r110	mTAS2r110		rTAS2r110	mTAS2r110		rTAS2r110	mTAS2r110		rTAS2r110	mTAS2r110		rTAS2r110	mTAS2r110
	rTAS2r113	mTAS2r113		rTAS2r113	mTAS2r113		rTAS2r113	mTAS2r113		rTAS2r113	mTAS2r113		rTAS2r113	mTAS2r113
	rTAS2r115	mTAS2r115		rTAS2r115	mTAS2r115		rTAS2r115	mTAS2r115		rTAS2r115	mTAS2r115		rTAS2r115	mTAS2r115
	rTAS2r116	mTAS2r116		rTAS2r116	mTAS2r116		rTAS2r116	mTAS2r116		rTAS2r116	mTAS2r116		rTAS2r116	mTAS2r116
	rTAS2r117	mTAS2r117		rTAS2r117	mTAS2r117		rTAS2r117	mTAS2r117		rTAS2r117	mTAS2r117		rTAS2r117	mTAS2r117
	rTAS2r123	mTAS2r123		rTAS2r123	mTAS2r123		rTAS2r123	mTAS2r123		rTAS2r123	mTAS2r123		rTAS2r123	mTAS2r123
	rTAS2r125	mTAS2r125		rTAS2r125	mTAS2r125		rTAS2r125	mTAS2r125		rTAS2r125	mTAS2r125		rTAS2r125	mTAS2r125
	rTAS2r129	mTAS2r129		rTAS2r129	mTAS2r129		rTAS2r129	mTAS2r129		rTAS2r129	mTAS2r129		rTAS2r129	mTAS2r129
	rTAS2r140	mTAS2r140		rTAS2r140	mTAS2r140		rTAS2r140	mTAS2r140		rTAS2r140	mTAS2r140		rTAS2r140	mTAS2r140

Stomach			Duodenum			Jejunum			Ileum			Colon		
Human	Rat	Mice	Human	Rat	Mice	Human	Rat	Mice	Human	Rat	Mice	Human	Rat	Mice
T2R16	rTAS2r118	mTAS2r118	T2R16	rTAS2r118	mTAS2r118	T2R16	rTAS2r118	mTAS2r118	T2R16	rTAS2r118	mTAS2r118	T2R16	rTAS2r118	mTAS2r118
	rTAS2r134	mTAS2r134		rTAS2r134	mTAS2r134		rTAS2r134	mTAS2r134		rTAS2r134	mTAS2r134		rTAS2r134	mTAS2r134
	rTAS2r143	mTAS2r143		rTAS2r143	mTAS2r143		rTAS2r143	mTAS2r143		rTAS2r143	mTAS2r143		rTAS2r143	mTAS2r143
T2R19	rTAS2r136 and rTAS2r120	mTAS2r136 and mTASr120	T2R19	rTAS2r136 and rTAS2r120	mTAS2r136 and mTASr120	T2R19	rTAS2r136 and rTAS2r120	mTAS2r136 and mTASr120	T2R19	rTAS2r136 and rTAS2r120	mTAS2r136 and mTASr120	T2R19	rTAS2r136 and rTAS2r120	mTAS2r136 and mTASr120
T2R20			T2R20			T2R20			T2R20			T2R20		
T2R30			T2R30			T2R30			T2R30			T2R30		
T2R31			T2R31			T2R31			T2R31			T2R31		
T2R38	rTAS2r138	mTAS2r138	T2R38	rTAS2r138	mTAS2r138	T2R38	rTAS2r138	mTAS2r138	T2R38	rTAS2r138	mTAS2r138	T2R38	rTAS2r138	mTAS2r138
T2R39	rTAS2r139	mTAS2r139	T2R39	rTAS2r139	mTAS2r139	T2R39	rTAS2r139	mTAS2r139	T2R39	rTAS2r139	mTAS2r139	T2R39	rTAS2r139	mTAS2r139
T2R40	rTAS2r144	mTAS2r144	T2R40	rTAS2r144	mTAS2r144	T2R40	rTAS2r144	mTAS2r144	T2R40	rTAS2r144	mTAS2r144	T2R40	rTAS2r144	mTAS2r144
T2R41	rTAS2r126	mTAS2r126	T2R41	rTAS2r126	mTAS2r126	T2R41	rTAS2r126	mTAS2r126	T2R41	rTAS2r126	mTAS2r126	T2R41	rTAS2r126	mTAS2r126
T2R42	rTAS2r145	mTAS2r131	T2R42	rTAS2r145	mTAS2r131	T2R42	rTAS2r145	mTAS2r131	T2R42	rTAS2r145	mTAS2r131	T2R42	rTAS2r145	mTAS2r131
T2R43	rTAS2r136 and rTAS2r120	mTAS2r136 and mTASr120	T2R43	rTAS2r136 and rTAS2r120	mTAS2r136 and mTASr120	T2R43	rTAS2r136 and rTAS2r120	mTAS2r136 and mTASr120	T2R43	rTAS2r136 and rTAS2r120	mTAS2r136 and mTASr120	T2R43	rTAS2r136 and rTAS2r120	mTAS2r136 and mTASr120
T2R45			T2R45			T2R45			T2R45			T2R45		
T2R46			T2R46			T2R46			T2R46			T2R46		
T2R50			T2R50			T2R50			T2R50			T2R50		
T2R60	rTAS2r135	mTAS2r135	T2R60	rTAS2r135	mTAS2r135	T2R60	rTAS2r135	mTAS2r135	T2R60	rTAS2r135	mTAS2r135	T2R60	rTAS2r135	mTAS2r135
–	rTAS2r137	mTAS2r137	–	rTAS2r137	mTAS2r137	–	rTAS2r137	mTAS2r137	–	rTAS2r137	mTAS2r137	–	rTAS2r137	mTAS2r137
?	?	mTAS2r122	?	?	mTAS2r122	?	?	mTAS2r122	?	?	mTAS2r122	?	?	mTAS2r122

Cells shaded green show that the expression is reported, those shaded red shows that no expression is reported, those shaded white show that no data are available, ? means that there are no orthologs in the species and – means that no information exists on the presence of the ortholog in that species. Data are obtained from (1, 22, 36, 49–56).

It has been reported that that TAS2R groups were present before the divergence of the primate and rodent lineages. However, differences in subsequent evolutionary processes led to species-specific sequences and shaped the diversity of the current TAS2R receptor families (58). In agreement with this, our analysis reveals that the three species do not follow the same expression pattern. Very few receptors are reported to be expressed in all the species: these are T2R4/Tas2r108 in almost all tissues, T2R38/Tas2r138 in the duodenum, and T2R1/Tas2r119 in the colon (1, 36, 49, 52, 53).

Taking into account currently available data, if we compare humans and rodents we observe that some receptors are maintained in humans but diverged in rodents (e.g., T2R10 or T2R14) (4). Whereas T2R14 is expressed throughout the intestine in humans, not all the orthologs are expressed in rodents. Moreover, in the duodenum, jejunum and ileum of rodents only one or no receptor is expressed. This type of expression pattern is also seen in individual receptors such as T2R4/mTas2r108, T2R38/mTas2r138, T2R42/mTas2r131, and T2R60/mTas2r135 (49, 52, 53, 56). On the other hand, some receptors, such as Tas2r119, Tas2r134, Tas2r143, Tas2r135, and Tas2r126, are expressed in both rodent species but not in humans (36, 51). The remaining expressed receptors, which are highest in number, are expressed only in one species. These include multiple receptors in mice, rats and humans in all intestinal tissues. On the whole, humans have the lowest number of such receptors expressed.

As stated earlier, the amount of data available in the literature on these three species varies considerably since analyses of rodent expression are scarce. On the other hand, since the available human datasets cover every identified TAS2R, it is easier to obtain more reliable results and, in agreement with other reports, we found that humans present a lower comparative number of TAS2R than rodents. It has been suggested that positive selection, which provides a diverse repertoire of bitter-tasting binding receptors in rodents (mainly mice), may account for the greater diversity in the TAS2R family during their evolution. Our analysis strengthens the idea that, as well as being a central warning pathway against the ingestion of potentially toxic substances, the expression of bitter taste receptors is mainly related to the specialization of organisms to different diets and the detection of molecules responsible for their activation (58).

In further studies it would be interesting to determine which receptors are activated by similar ligands in order to establish functional orthologs between species. Moreover, expression analysis on the intestinal tissue of rats should be carried out so as to expand the available information. Further studies are needed to find possible differences in the activation pathway between oral and extraoral receptors and discover the role of these extraoral receptors in each intestinal segment.

## Author contributions

MD-S and AV contributed to design of the review and wrote the first draft of the manuscript. FJ wrote sections of the manuscript. XT and RB-D provided critical feedback. AA conceived the original idea, provided critical feedback, and leadership responsibility for the research activity planning and execution. MB, ER-G, and MP contirbuted to the conception of the inicial idea, funding provision and critical review. All authors contributed to the article and approved the submitted version.

## Funding

This research was funded by Grant PID2021-122636OB-I00 funded by MCIN/AEI/10.13039/501100011033/ and “ERDF A way of making Europe.” Martí Franqués program of Universitat Rovira i Virgili. FISDUR and Serra Húnter fellows from Generalitat de Catalunya. This project has received funding from the European Union’s Horizon 2020 research and innovation programme under the Marie Skłodowska Curie grant agreement no. 945413 and from the Universitat Rovira i Virgili (URV).

## Conflict of interest

The authors declare that the research was conducted in the absence of any commercial or financial relationships that could be construed as a potential conflict of interest.

## Publisher’s note

All claims expressed in this article are solely those of the authors and do not necessarily represent those of their affiliated organizations, or those of the publisher, the editors and the reviewers. Any product that may be evaluated in this article, or claim that may be made by its manufacturer, is not guaranteed or endorsed by the publisher.

## Footnotes

^1^ https://rgd.mcw.edu

^2^ https://www.informatics.jax.org

^3^ http://www.proteinatlas.org
